# Spuriously Elevated Serum IGF-1 in Adult Individuals with Delayed Puberty: A Diagnostic Pitfall

**DOI:** 10.1155/2010/370692

**Published:** 2010-08-30

**Authors:** Syed Ali Imran, Michael Pelkey, David B. Clarke, Dale Clayton, Peter Trainer, Shereen Ezzat

**Affiliations:** ^1^Division of Endocrinology & Metabolism, Dalhousie University, Halifax, NS, Canada B3H 3J5; ^2^Divisions of Endocrinology & Metabolism & Neurosurgery, Halifax Neuropituitary Program, 7th Floor N, VG Site, 1278 Tower Road, Halifax, NS, Canada B3H 2Y9; ^3^Division of Endocrinology, The University of Manchester, Manchester M13 9PL, UK; ^4^Division of Endocrinology, University of Toronto, Toronto, ON, Canada M5S 1A1

## Abstract

Serum insulin-like growth factor-1 (IGF-1) is a sensitive marker of growth hormone (GH) activity. The levels of IGF-1 vary widely, peaking during puberty and declining with advancing age. During adolescence, serum IGF-1 levels tend to correlate better with pubertal stage rather than chronological age. Here we discuss two cases of delayed puberty, both in their 20s, who presented with high serum IGF-1 but no clinical or biochemical evidence of hypersomatotropism as confirmed by appropriate GH response to an oral glucose challenge. Both individuals achieved full pubertal status with testosterone replacement therapy and their serum IGF-1 levels settled into normal age-specific range. We suggest that in chronologically adult individuals with delayed puberty, serum IGF-1 should not be interpreted on the basis of age-specific normal values but rather on their pubertal status. Furthermore, in the absence of another cause of elevated IGF-1, the expectation is that IGF-1 levels will decline towards age-normative ranges following androgen replacement therapy.

## 1. Introduction

The measurement of serum insulin-like growth factor-1 (IGF-1) is a useful screening test for acromegaly [1]. The normal reference range for IGF-1 varies widely, peaking during puberty and declining with advancing age [2]. With the onset of puberty, the release of growth hormone (GH) is stimulated through sex steroids with estrogen being the main stimulator of GH release both in males and females [3]. Although in males testosterone also stimulates the release of GH, the majority of the GH effect still occurs through aromatization of testosterone to estrogen [4]. Once puberty has been completed, the levels of serum IGF-1 drop by fifty percent during early adulthood and then continue to decline throughout adult life [2]. Therefore, serum IGF-1 levels are interpreted against age and gender-specific reference ranges. Here we describe 2 cases of delayed puberty who presented with elevated IGF-1 levels that normalized with androgen replacement therapy.

**Case 1**

A 21-year-old male university student was referred to our centre for bilateral gynecomastia and delayed puberty. He reported minimal pubic hair development at the age of 16, absent sense of smell, and inability to ejaculate. He had undergone varicocele surgery at age 15. On clinical examination, he was 194 cm tall with a body mass index (BMI) of 22 Kg/m^2^, and had an arm span of 202 cm. His visual fields were normal, and there were no midline defects. The testicular volumes were 5 and 8 mLs for right and left testes, respectively, with an infantile phallus, graded as Tanner stage 2. Serum total testosterone was low on two separate occasions at 1.0 and 3.4 nmol/L (normal range for adult: 8.4–28.7 and for Tanner stage 2: 0.6–5.3), and serum follicle stimulating hormone (FSH) and luteinizing hormone (LH) levels were 0.4 and 0.3 IU/L, respectively, (normal ranges: 1.4–18.1 IU/L and 1.5–9.3 IU/L, resp.) thus confirming secondary hypogonadism. The remaining pituitary function tests, including serum thyroid stimulating hormone (TSH), free T4, morning cortisol, and prolactin, were normal. His serum IGF-1 was elevated to 485 and 675 ug/L on two separate occasions (normal range for males age 21–25 years: 116–358 ug/L. (All IGF-1 estimations were performed using Siemens Immulite 2500 chemiluminescent assay (Siemens, IL, USA). The normal reference ranges are stratified by 1-year intervals until age 20 and then 5-year intervals until age 85.) The 5- hour oral glucose tolerance test showed appropriate suppression of growth hormone to less than 1 ug/L, and MRI did not reveal any abnormality in the hypothalamic-pituitary region. He was clinically diagnosed as having Kallmann's syndrome and started on testosterone replacement therapy in the form of testosterone enanthate injections. Three years later, he had normal serum total testosterone and he had achieved Tanner stage 5 pubertal development. His BMI remained unchanged and his IGF-1 normalized at 312 ug/L.

**Case 2**

A 22-year-old male presented with secondary hypogonadism as a result of investigation by his general practitioner for loss of libido, erectile dysfunction, and fatigue. His history was unremarkable for any cause of hypogonadism. On examination, he was 170 cm tall and weighed 113 kg with a BMI of 39.1 kg/m^2^. He was Tanner stage 4 for both pubic hair and genital development, with 12–15 mL testes bilaterally. Initial biochemical testing showed a total testosterone of 6.1 nmol/L (normal range for adult: 8.4–28.7 and for Tanner stage 4: 7.0–21.7) with an undetectable LH and FSH. Repeat testing 3 months later showed a total testosterone of 3.2 nmol/L, with an FSH of 0.6 IU/L and LH of 0.1 IU/L. Serum morning cortisol, TSH, free T4, and prolactin levels were normal; however, serum IGF-1 was above reference range at 405 ug/L (normal range for males age 21–25 years : 116-358 ug/L. (All IGF-1 estimations were performed using Siemens Immulite 2500 chemiluminescent assay (Siemens, IL, USA). The normal reference ranges are stratified by 1-year intervals until age 20 and then 5-year intervals until age 85.) The oral glucose tolerance testing was normal, demonstrating GH suppression to less than 1 ug/L. MRI of the sella revealed a normal pituitary with no anatomical abnormalities in the hypothalamic-pituitary region. In view of the biochemical evidence of hypogonadism, he was started on intramuscular testosterone enanthate replacement therapy which normalized his serum total testosterone. One year later, he achieved Tanner stage 5 puberty, his BMI remained unchanged, and his IGF-1 normalized to 258 ug/L.

## 2. Discussion

The two cases presented here highlight an association between delayed puberty due to central hypogonadism and elevated IGF-1 levels. In both cases, these individuals did not have clinical or biochemical evidence of hypersomatotropism, as confirmed by an appropriate GH response to an oral glucose challenge, and their IGF-1 levels normalized within 12–36 months of starting androgen replacement therapy.

Measurement of serum IGF-1 is a sensitive marker of GH activity, and, based on recent guidelines, a low serum IGF-1 in patients with a history of organic pituitary disease confirms growth hormone deficiency whereas an elevated IGF-1 is regarded as the best single test for the diagnosis of acromegaly [5]. The circulating IGF-1 is mostly produced in the liver with GH being the primary regulator of serum IGF-1 levels. In addition to GH, several other factors modulate serum IGF-1 concentration. For instance, reduced caloric intake and conditions associated with malnutrition such as inflammatory bowel disease, renal failure, and hepatic failure are associated with low IGF-1 levels [6, 7]. On the contrary, serum IGF-1 levels may be elevated during pregnancy primarily due to the highly somatogenic effect of placental GH which mostly replaces circulating pituitary GH [8]. The association between obesity and IGF-1 is controversial with some studies reporting high [9, 10] while others reporting low IGF-1 levels [11] in association with obesity. The pubertal rise in IGF-1 levels reflects an increase in GH secretion at puberty [12] and the effect of gonadal steroids which increase GH concentration by increasing the growth hormone releasing hormone pulse amplitude [4]. Serum IGF-1 levels are evaluated against age- and gender-adjusted normative values. These values are low at birth, peak during late puberty, fall to about half maximal concentrations in the second to third decades, and continue to decline as aging occurs [2]. During adolescence, IGF-1 levels tend to correlate better with the Tanner stage of puberty as opposed to chronological age, peaking quickly by Tanner stage 3-4 in girls and Tanner stage 4 in boys [13, 14]. In our patients the normal reference ranges for serum IGF-1 based on their Tanner stages were 67–414 ug/L (Case 1) and 187–854 ug/L (Case 2). In Case 1, serum IGF-1 was higher than both age and Tanner stage-specific ranges whereas in Case 2 it was above the age-specific range but within the Tanner stage-specific normal range. These two individuals were both in their 20s and, therefore, well past the adolescent age group. However, they had failed to achieve puberty until appropriate androgen replacement therapy was started. Retrospectively, the elevated IGF-1 levels in these patients were more congruent with their Tanner stages rather than chronological age and these levels normalized when they received androgen replacement therapy. There are conflicting reports about the association between sex steroids and serum IGF-1 levels during pubertal years. While some studies have suggested significant correlation between sex steroids and IGF-1 levels in male and female adolescents [15, 16], others have reported such association only in girls and not in boys [17]. Furthermore, serum IGF-1 levels are notably elevated in adolescents with precocious puberty where they normalize after suppression of sex steroids with gonadotropin-releasing hormone therapy [18]. Interestingly some studies have also reported that in both hypogonadotropic females and males, and boys with constitutional delay in puberty, treatment with respective sex steroids increases serum IGF-1 [19–21]. Previously another study of prepubertal hypopituitary boys treated with testosterone therapy for eight days reported no difference in the response of IGF-1 to GH challenge [22]. Contrary to these reports, serum IGF-1 levels were elevated in our patients in keeping with their pubertal status and normalized with androgen replacement therapy. Despite demonstrating a different response of serum IGF-1 to androgen replacement therapy from the previously published data, our observation lends support to the evidence that a definite association exists between sex steroids and serum IGF-1 levels during pubertal period. It is difficult to speculate the reason for this disparity from other published reports, but it may be due to the underlying pathology, duration of treatment, and the age at assessment.

As noted earlier, unlike previously reported studies our patients were both in their 20s, an age where such individuals would normally see adult endocrinology. Most adult endocrinology clinics tend to use age-based reference values for IGF-1; therefore, even in this age group, such a disparity could potentially lead to diagnostic misinterpretation. These cases indicate that in young adults with delayed puberty, serum IGF-1 estimation should not be based on age specific normative values but instead should be interpreted in the context of Tanner stage. Furthermore, in the absence of another cause of elevated IGF-1, the expectation is that IGF-1 levels will decline towards age-normative ranges following androgen replacement therapy.

## 3. Conclusion

We describe cases of two adult males with delayed puberty secondary to hypogonadism whose IGF-1 levels were elevated for their age-specific normal reference ranges. In both cases, these values normalized once puberty with androgen replacement therapy was achieved. Therefore, in chronologically adult individuals with delayed puberty, serum IGF-1 assessment should not be based on age-specific normal values but rather on their Tanner stage of puberty. Since most reference laboratories currently report serum IGF-1levels based solely on age- and gender-based normative values, additional availability of such reference ranges based on pubertal stage would avoid diagnostic confusion.
